# Editorial: Updates on combination therapy for lung cancer volume II

**DOI:** 10.3389/fonc.2024.1393278

**Published:** 2024-04-19

**Authors:** Alberto Pavan, Liyun Shi, Muhammad Abbas

**Affiliations:** ^1^ Medical Oncology, Azienda Unità Locale Socio-Sanitaria (ULSS) 3 Serenissima, Venice, Italy; ^2^ Institute of Translational Medicine, Zhejiang Shuren University, Hangzhou, Zhejiang, China; ^3^ Riphah Institute of Pharmaceutical Sciences, Riphah International University, Islamabad, Pakistan

**Keywords:** NSCLC, SCLC, immunotherapy, anti-angiogeneic therapy, computational biology

Lung cancer is still one of the leading causes of death, representing the most common cancer in both sexes, with over 2.21 million new cases diagnosed yearly (1). The prognosis of lung neoplasms is often poor due to the late diagnosis, which leaves space only for palliative treatment, and due to the molecular and histological heterogeneity of the disease (2).

Despite such a dismal background, several advancements have occurred during the last decade. On one hand, the advent of an entirely new class of drugs, the immune-checkpoint inhibitors (ICIs)—capable of tackling tumor cells and unleashing the killing mechanisms of a patient’s immune system, has radically improved the survival rates (3). On the other hand, a wider molecular characterization of lung cancer cells and their ability to interact with their surrounding has led to the development of targeted agents and new treatment strategies. The scientific community is constantly striving to identify new molecular pathways to exploit using new anti-cancer agents. However, as a direct consequence of this decade of improvement in lung cancer care, we are facing a new challenge: the need to build the best treatment sequence for each patient, integrating different therapies that, over time, have all proved to be effective.

This Research Topic encompasses several articles that address both these crucial challenges. With regards to the “treatment integration” issue, the works of Garon et al. and Yang et al. deal with the role of anti-angiogenic drugs. Angiogenesis plays an important role not only in tumor growth, invasion, and metastasis but also in the acquired resistance to immunotherapy (4). In fact, the Vascular Endothelial Growth Factor (VEGF) is able to exert an immunosuppressive effect on the tumor microenvironment, stimulating the recruitment of immunosuppressive cells and blocking the antigen-presenting process by inhibiting dendritic cell maturation (5). Anti-angiogenic agents proved their efficacy in lung cancer care in a pre-immunotherapy scenario; the advent of ICIs in the first-line setting has created a certain degree of doubt regarding their true impact when administered at disease progression for immunotherapy. In their systematic review, Garon et al. collected all the available evidence on the efficacy and safety of ramucirumab plus docetaxel regimen in an ICI pretreated and in an ICI-naïve group of patients, respectively (Garon et al.). This review confirmed the potential of this regimen also in ICI-pretreated patients, especially in terms of improved progression free survival (PFS) and of higher overall response rate. On the other hand, Yang et al.‘s original research article evaluated the efficacy of another antiangiogenic drug, recombinant human (rh) endostatin, in combination with ICI, as a second-line treatment for advanced non-small cell lung cancer (NSCLC). The combination resulted in a significant improvement both in terms of PFS and in terms of overall survival (OS). These data confirm the synergism between these two classes of drugs, consolidating the role of antiangiogenics in the treatment sequence, especially for pre-treated patients, as suggested in some early phase trials (6, 7).

Treatment patterns have changed also in the field of SCLC care. Here, the introduction of chemo-immunotherapy in clinical practice surely had a smaller impact compared with the NSCLC experience; however, as reported in phase IV and in several real-world studies, a subgroup of patients seems to benefit from such strategy over longer periods (8–10). Interestingly, the real-world study by Zheng et al., included in this Research Topic, confirmed the data coming from phase III trials in terms of survival benefit but also pointed out the presence of a subgroup of long-term surviving patients benefiting from first-line chemo-immunotherapy and less likely to need further lines of treatment. Such a result is impressive for a disease that is so challenging to treat that it was officially defined as a “recalcitrant cancer” (11). Unluckily, there is still an unmet need regarding predictive tools able to identify patients who would benefit longer from first-line chemo-immunotherapy (12). Moreover, the work of Zheng et al. also shed light on the number of drug regimens that might have a role for pre-treated patients. In this field, the number of treatment options is rapidly growing, as completely new classes of agents are showing some interesting results in terms of efficacy and survival impact (13, 14).

Finally, regarding the issue of identifying new targets to exploit in cancer treatment, this Research Topic includes the fascinating work of Altaf and colleagues (15). Here, the authors used computational biology to narrow the perimeter of research for new targets. NSCLC-associated differentially expressed genes were firstlyfirst obtained from different datasets and subsequently validated at the translational level. Furthermore, a drug–gene network analysis was performed in order to correlate the identified genes with agents capable of significant interactions. This approach has the potential to speed up the drug-discovery process, while reducing the related costs (16, 17) and designing tailored therapeutic approaches.

Altogether, the original articles and reviews in this Research Topic represent an invaluable resource of insights on into important achievements attained in the field of lung cancer care, as well as strategies for the delivery of precision medicine approaches in thoracic oncology.

## Author contributions

AP: Writing – original draft, Writing – review & editing. LS: Writing – review & editing. MA: Writing – review & editing.
